# Deferred

**Published:** 1884-01

**Authors:** 


### DEFERRED.

The first part of Dr. Miller's promised article has been received, but as it is impossible to get the necessary cuts made in season, its publication is necessarily delayed until the February number.

